# Web-Based Smartphone Algorithm for Calculating Blood Pressure From Photoplethysmography Remotely in a General Adult Population: Validation Study

**DOI:** 10.2196/19187

**Published:** 2021-04-23

**Authors:** Paul Holyoke, Karthika Yogaratnam, Elizabeth Kalles

**Affiliations:** 1 SE Research Centre SE Health Markham, ON Canada

**Keywords:** blood pressure measurement, remote monitoring, hypertension

## Abstract

**Background:**

Outside of a clinical setting, oscillometric devices make remote monitoring of blood pressure and virtual care more convenient and feasible. HeartBeat Technologies Ltd developed a novel approach to measuring blood pressure remotely after an initial blood pressure reading by a nurse using the conventional measurement method. Using a finger pulse oximeter, a photoplethysmogram wave is transmitted by Bluetooth to a smartphone or tablet. A smartphone app (MediBeat) transmits the photoplethysmogram to a server for analysis by a proprietary algorithm—the person’s current blood pressure is sent back to the smartphone and to the individual’s health care provider.

**Objective:**

This study sought to determine whether the HeartBeat algorithm calculates blood pressure as accurately as required by the European Society of Hypertension International Protocol revision 2010 (ESH-IP2) for validation of blood pressure measuring devices.

**Methods:**

ESH-IP2 requirements, modified to conform to a more recent international consensus statement, were followed. The ESH-IP2 establishes strict guidelines for the conduct and reporting of any validation of any device to measure blood pressure, including using the standard manual blood pressure instrument as a comparator and specific required accuracy levels for low, medium, and high ranges of blood pressure readings. The consensus statement requires a greater number of study participants for each of the blood pressure ranges. The validation of the accuracy of the algorithm was conducted with a Contec CMS50EW pulse oximeter and a Samsung Galaxy XCover 4 smartphone.

**Results:**

The differences between the HeartBeat-calculated and the manually measured blood pressures of 62 study participants did not meet the ESH-IP2 standards for accuracy for either systolic or diastolic blood pressure measurements. There was no discernible pattern in the inaccuracies of the HeartBeat-calculated measurements.

**Conclusions:**

The October 4, 2019 version of the HeartBeat algorithm, implemented in combination with the MediBeat app, a pulse oximeter, and an Android smartphone, was not sufficiently accurate for use in a general adult population.

**Trial Registration:**

ClinicalTrials.gov NCT04082819; http://clinicaltrials.gov/ct2/show/NCT04082819

## Introduction

Accurately assessing blood pressure is necessary for the proper diagnosis of hypertension, implementing treatments and monitoring whether those treatments are working [1,2]. To measure blood pressure accurately, standardized measurement techniques, calibrated equipment, and valid interpretation of readings are necessary [2-4]. Aneroid sphygmomanometers that are properly calibrated and maintained are considered to be at least as accurate as mercury-filled sphygmomanometers [5]. However, such instruments are not convenient for remote monitoring or virtual (remote) care because they can be difficult for a person to self-administer.

A novel approach to measuring blood pressure remotely has been developed (HeartBeat, HeartBeat Technologies Ltd). The measurement is taken after an initial blood pressure reading using the conventional measurement method, supplemented by specific characteristics of a person (age, gender, height, weight, and heart rate) to establish a baseline for the person. Unlike other devices [6-9], this approach uses a finger pulse oximeter that detects the changes in blood volume directly below the person’s skin and indirectly measures oxygen saturation in the blood. The resulting photoplethysmogram (PPG) wave is transmitted by Bluetooth technology to a smartphone or tablet. The MediBeat (HeartBeat Technologies Ltd) smartphone or tablet app then transmits the PPG wave to a server where a proprietary algorithm analyzes the baseline measurement for the person and the PPG waveform to calculate the person’s current blood pressure. The blood pressure measurement is then transmitted to the person’s smartphone, and if applicable, to the person’s health care provider’s devices.

The purpose of this study was to test whether the accuracy of the HeartBeat algorithm, available as of October 4, 2019 and for a general adult population (ages 25 years and older), was sufficient to seek regulatory approval when compared with a standard aneroid sphygmomanometer with stethoscope for measuring blood pressure.

## Methods

### Protocol

The European Society of Hypertension International Protocol revision (ESH-IP2) for the validation of blood pressure measuring devices in adults [10] was followed, with modifications to account for additional guidance from an international consensus statement [11]. ESH-IP2 [10] requires strict adherence to its method and reporting requirements, which are reflected in this report: to “ensure a uniform distribution of test pressures across a representative range,” 33 participants must be enrolled. Among the 33 participants, at least 10 must be male and 10 must be female; all should be at least 25 years of age; and between 10 and 12 participants must have blood pressure readings within each of the following recruitment ranges—systolic blood pressure: below 130 mmHg, between 130 and 180 mmHg, above 180 mmHg; diastolic blood pressure: below 80mmHg, between 80 and 130 mmHg, above 130 mmHg. All potential participants must be screened against these criteria and can be excluded if the gender criteria or age criteria are not met, or if including them in the study would exceed the maximum number of participants in any of the recruitment ranges.

An international consensus statement group [11] reviewed the adequacy of the number of participants in a variety of established protocols, including that of the ESH-IP2, and recommended that, to increase the power and accuracy of validation studies, at least 85 participants, rather than 33, should be included. To align with the consensus statement, the research team recalculated the ESH-IP2 requirement numbers to reflect the increased participant pool of 85. The recalculations are reflected in the tables and discussion in this report.

### Devices

**Figure 1:**
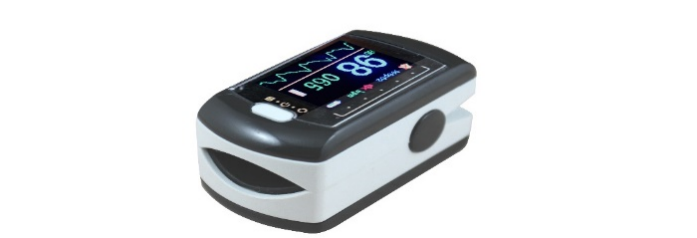
Contec CMS50EW.

**Figure 2:**
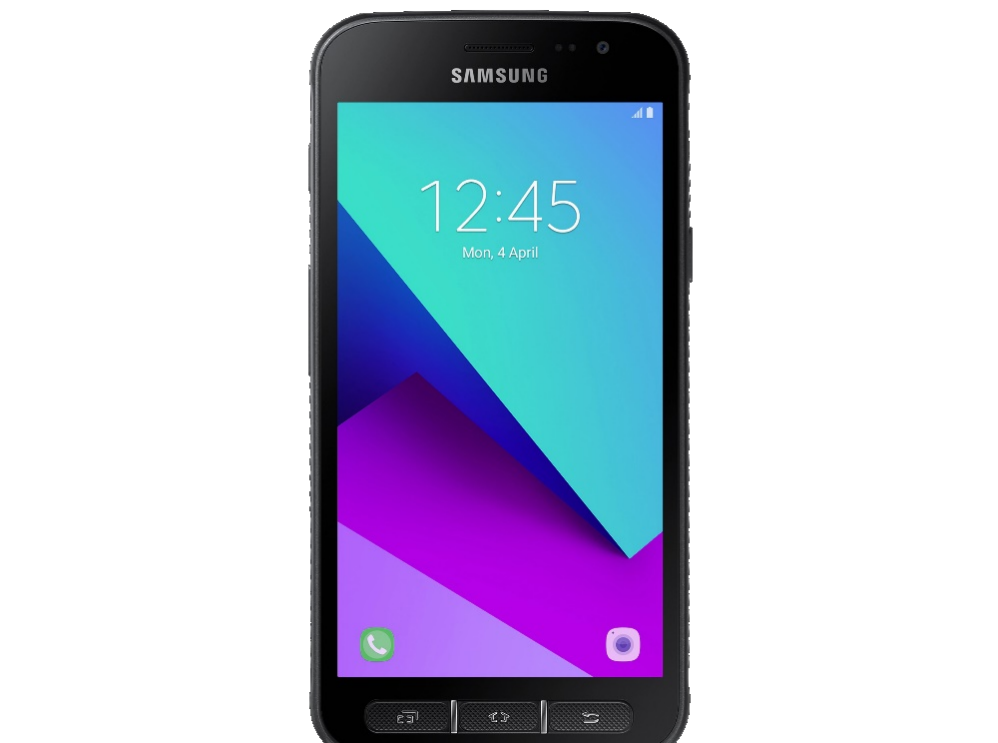
Samsung Galaxy XCover 4.

The pulse oximeter (Contec CMS50EW, Contec Medical Systems Co Ltd [12]) used to capture and transmit PPG is shown in Figure 1. The 8-bit Contec pulse oximeter is approved for use by Health Canada [13] and the US Food and Drug Administration [14]. The smartphone used in this study (upon which HeartBeat’s MediBeat app was installed and used to transmit the data to HeartBeat servers and to receive and display the results) was a Samsung Galaxy XCover 4 (Figure 2).

### Recruitment

Potential participants were recruited by mass email and offline posters at a single-site multistory office building in Markham, Ontario, Canada, where they worked; participants provided informed consent (Multimedia Appendix 1) and were scheduled for a measurement appointment during their working day by a member of the research team.

### Procedure

Three registered nurses who had training and expertise in blood pressure measurement carried out the measurements required for this study between October 4, 2019 to November 22, 2019. One nurse was the supervisor, and two nurses were the observers throughout the duration of the study. The nurses were trained as per ESH-IP2 directives and were instructed on how to follow the protocol guidelines for data collection. Blood pressure measurements were recorded to the nearest 2 mmHg.

Two new sphygmomanometers (Prosphyg 775 Model, American Diagnostics Corporation [15]), whose components were checked against one another and calibrated before the study, were used as the reference instruments by the observers. With respect to reliability and validity, the ESH-IP2 embeds an ongoing reliability mechanism requiring that any individual participants’ blood pressure readings be excluded from the analysis if the difference in readings from the two observer nurses differ by 4 mmHg twice. The instruments were placed within one meter of the observers while they interacted with a participant, which allowed the observers to follow the instruments’ dials at eye level from 40 mmHg to 180 mmHg. Multiple sizes of bladders (cuffs) were available for the sphygmomanometers to ensure that 80% to 100% of each participant’s arm circumference could be encircled. The observers were also supplied with good quality nonelectronic stethoscopes with well-fitted earpieces.

The observers were instructed that, in the event that an abnormal blood pressure reading was detected (for example, if the blood pressure was outside clinically normal range for a specific person), they were to remeasure the blood pressure using conventional means to determine if the abnormal reading was accurate. If the abnormal blood pressure measurement was accurate, the nurse supervisor was to act according professional standards. The abnormal blood pressure measurement should also be recorded on ESH-IP2 Form 2.

Participants’ blood pressure measurements were taken in a room that was a comfortable temperature, without any noises or other influences that would have caused disturbances, such as telephones. The temperature in the room was the same as that throughout the building—20 °C to 24 °C. All participants participated in the study between the working hours of 9 AM and 4:30 PM. The pulse oximeter and the supervisor’s smartphone were charged and tested each day prior to interactions with participants. The MediBeat app was calibrated for each participant as follows: (1) Reference data for the individual (year of birth, height, weight, baseline manual blood pressure reading, and heart rate) were entered. (2) The participant rested for 10 minutes while seated comfortably with their legs uncrossed, back supported, and arm resting on the table at heart level. (3) The supervisor attached the finger pulse oximeter to the participant’s index finger (on the opposite arm to the manual blood pressure cuff) and waited for a few seconds until a stable signal was received and the device displayed both the participant’s pulse and SpO_2_ (oxygen saturation) values. (4) Once the values were displayed, the supervisor initiated a new session on the smartphone to connect the phone to the Contec device via Bluetooth. This connection started the 1-minute calibration process, at which point each participant was asked to remain still until the calibration had been completed successfully.

**Figure 3:**
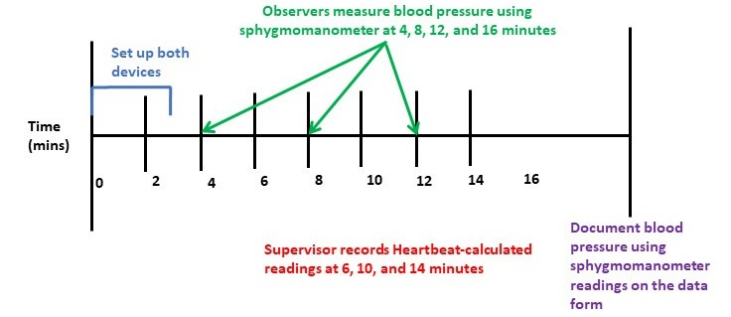
Timeline of measurement of blood pressure for each participant.

Overseen by an independent supervisor, measurements were recorded by the two observers who were blinded from both the other observer’s readings and from the reading calculated by the HeartBeat algorithm. Each participant’s blood pressure was measured as illustrated in Figure 3. Each systolic blood pressure measurement and each diastolic blood pressure measurement calculated by the HeartBeat algorithm was compared with the nearest of the previous and next observer systolic and diastolic measurements, respectively, and the difference between each HeartBeat-calculated measurement and the nearest observer measurement was calculated. Every such difference was then classified in one of these error groups for each systolic and diastolic measurements: 0-5 mmHg, 6-10 mmHg, 11-15 mmHg, and >15 mmHg. The results were then compared with the number of tolerable differences allowed by the protocol at both the level of individual measurements (part 1) and at the level of the participants (part 2). The device gets an overall *pass* (an acceptable level of accuracy) if it meets 4 pass criteria.

### Ethics

The study was reviewed and approved by the Southlake Regional Health Centre Research Ethics Board, Newmarket, Ontario, Canada (007-1920).

## Results

**Figure 4:**
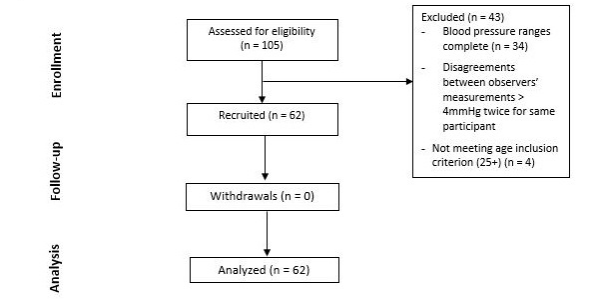
Adapted version of the CONSORT flow diagram [16].

**Figure 5:**
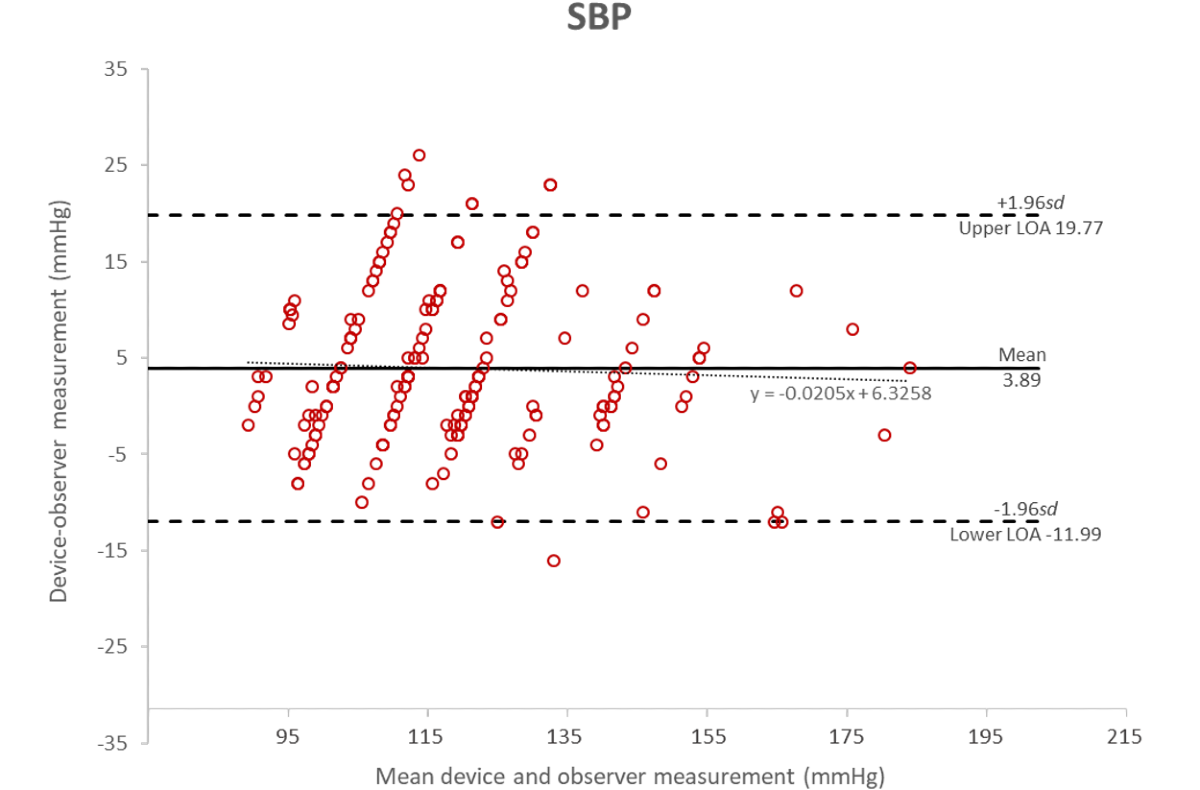
Bland-Altman plot of the differences between the Heartbeat-calculated measurements and the manual measurements of systolic blood pressure.

**Figure 6:**
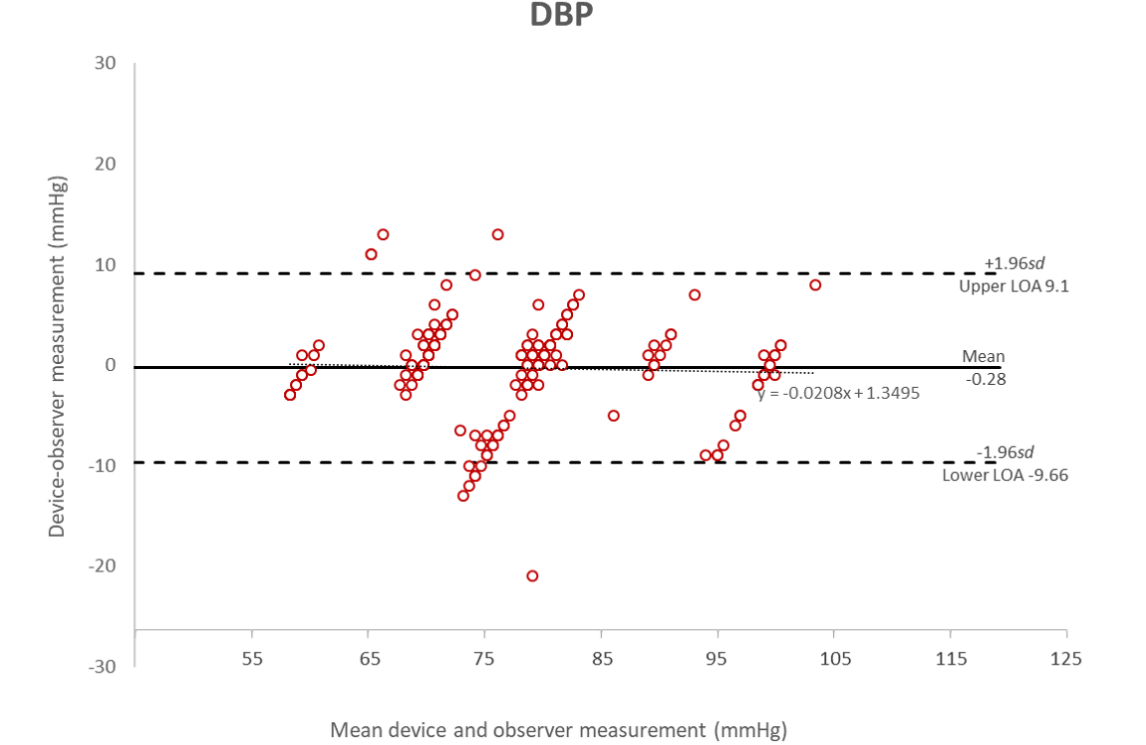
Bland-Altman plot of the differences between the Heartbeat-calculated measurements and the manual measurements of diastolic blood pressure.

A total of 105 individuals volunteered and were screened for the study, of whom 62 participants met the ESH-IP2 criteria, and 43 were excluded (Table 1; adapted version of the CONSORT flow diagram [16] in Figure 4). As required by the protocol, 5 participants out of the 105 were excluded because the differences in the measurements by the observer nurses exceeded 4 mmHg twice for the same participant, 34 were excluded participants were excluded because their blood pressure measurements fell within ranges for which the protocol quota had been fulfilled, and 4 participants were excluded because they were younger than 25 years of age. There was difficulty in recruiting hypertensive participants, that is, individuals with both systolic and diastolic blood pressures in the high ranges. This is reflected in the overall distribution (Figure 5 and Figure 6), in which most of the points fall below 125 mmHg and 90 mmHg, for systolic and diastolic blood pressures, respectively. By the time 105 participants had been recruited, only 2 met the criterion for high systolic blood pressure, and none met the criterion for diastolic blood pressure. The modified criteria would have required between 26 and 31 participants in the high blood pressure group to meet the protocol requirements. A calculation was performed to determine if it would be possible for the algorithm to achieve a pass if additional participants with high blood pressure were recruited, and there were a perfect match between their HeartBeat-calculated measurements and standard measurements. It was determined that such a result was not possible, and recruitment was discontinued. Therefore, the study concluded before the required number of participants was reached. The characteristics of participants are described in Tables 2 and 3.

**Table 1:** Participants screened, excluded, and recruited for the study.

Reason for exclusion			Excluded, n		Total, n
Participants screened					105
**Participants excluded**					43
	Ranges complete^a^	34			
	Range adjustment	0			
	Arrhythmias	0			
	Device failure	0			
	Poor quality sounds	0			
	Cuff size unavailable	0			
	Observer disagreement^b^	5			
	Distribution^c^	0			
	Other reasons^c^	4			
Participants recruited					62

^a^Intake assessment placed them in ranges that had already been filled. The ESH-IP2 requires that the last participants to be recruited are the ones who are excluded.

^b^Discrepancy >4 mmHg twice for the same participant; therefore, these participants were, in accordance with the ESH-IP2, excluded from the study.

^c^Age <25 years or older, as required by the ESH-IP2.

**Table 2:** Blood pressure and medication details for initially screened participants.

Category		Blood pressure (mmHg), range		Medication^a^		No medication		Total
**Systolic blood pressure**								
	Low-low	<90	0		0		0	
	Low	90-129	5		40		45	
	Medium	130-160	7		9		15	
	High	161-180	1		1		2	
	High-high	>180	0		0		0	
**Diastolic blood pressure**								
	Low-low	<40	0		0		0	
	Low	40-79	3		30		33	
	Medium	80-100	10		19		29	
	High	101-130	0		0		0	
	High-high	>130	0		0		0	

^a^Participants taking medication for hypertension.

**Table 3:** Participant details.

Category					Value
**Sex, n**					
	Male			13	
	Female			49	
**Age (years)**					
	Mean (SD)			43.5 (11.2)	
	Range			25-66	
**Arm circumference (cm)**					
	Mean (SD)			29.5 (4.2)	
	Range			23-40	
**Cuff size used, n**					
	Small			0	
	Standard			61	
	Large			0	
	Missing data			1	
**Recruitment blood pressure (mmHg)**					
	**Systolic blood pressure**				
		Mean (SD)	119.5 (18.9)		
		Range	90-180		
	**Diastolic blood pressure**				
		Mean (SD)	77.6 (10.6)		
		Range	57-110		

Table 4 and Table 5 present the blood pressure readings by the international consensus statement’s predetermined levels. Table 4 shows the distribution of systolic and diastolic blood pressure readings across the recruited participants in the study. Table 5 shows the range of differences by observers 1 and 2.

**Table 4:** Blood pressure readings (3 per participant) after the initial screening measurement.

	Overall range (mmHg) (low-high)	Low^a^, n	Medium^b^, n	High^c^, n	Maximum difference among highest and lowest totals in low, medium, and high ranges
Systolic blood pressure	90-180	150	30	6	144
Diastolic blood pressure	60-101	90	75	21	69

^a^Systolic ≤130 mmHg; diastolic ≤80 mmHg.

^b^Systolic 130 mmHg-160mmHg, diastolic 80 mmHg-100 mmHg.

^c^Systolic ≥160 mmHg, diastolic ≥100 mmHg.

**Table 5:** Observer differences.

	Range (low to high) (mmHg)	Mean (SD)	Repeated measurements
Systolic blood pressure^a^	–3 to 3	0.0081 (0.4)	—^b^
Diastolic blood pressure^a^	–4 to 3	0.0000 (0.6)	—^b^

^a^Measurements are calculated by subtracting observer 1 measurements from observer 2 measurements.

^b^There were no documented repeated measurements to calculate.

Table 6 shows the results in terms of each of the protocol requirements. According to the ESH-IP2 standards, for an acceptable level of accuracy, a device must pass all the protocol requirements in both parts 1 and 2. Note that in this study, the algorithm achieved a pass in only 1 category and a fail in 3 categories, for an overall fail.

**Table 6:** Validation results.

Protocol requirements				Range, n							Pass or fail
				<5 mmHg		<10 mmHg		<15 mmHg			
**Part 1 (N=186^a^)**											
	Required (2 of 3)		At least 137		At least 163		At least 180				
	**Achieved differences**										
		Systolic blood pressure^a^	120		147		169		Fail		
		Diastolic blood pressure^a^	159		180		186		Pass		
	Required (all)		At least 122		At least 152		At least 175				
	**Achieved differences**										
		Systolic blood pressure	120		147		169				
		Diastolic blood pressure	159		180		186				
**Part 2 (N=62^a^)**											
	Required		At least 45						Fail		
	**Achieved**										
		Systolic blood pressure	43		—^b^		—				
		Diastolic blood pressure	10		—		—				
	Allowed		At most 6						Fail		
	**Achieved**										
		Systolic blood pressure	54		—		—				
		Diastolic blood pressure	5		—		—				
Grade 3 (final result)—must pass all of parts 1 and 2										Fail	

^a^These reflect the number of readings and participants included in the study when recruitment was stopped.

^b^Not applicable.

Figure 5 shows the Bland-Altman plots [17,18] of the differences in systolic blood pressure measurements between the MediBeat device and the manual observer measurements (*y*-axis) and the average of the 2 measurements (*x*-axis). Figure 6 shows the plots for the differences in diastolic measurements. In both cases, the averages of the 3 MediBeat measurements were plotted against their absolute differences from the manual measurements for both systolic blood pressure (bias 3.89, LOA –11.98 to 19.77) and diastolic blood pressure (bias –0.28, LOA –9.66 to 9.09). Although values fall within the 95% confidence interval for both Bland-Altman plots, there is no statistically significant correlation between the HeartBeat-calculated blood pressure measurements and the manual measurements (systolic blood pressure: *r*=0.04, *P*=.53; diastolic blood pressure: *r*=.04, *P*=.54). This indicates that the HeartBeat-calculated blood pressure measurements are not sufficiently accurate. There was no discernible pattern in the inaccuracies in the HeartBeat-calculated measures; therefore, the source of the inaccuracies is not known.

## Discussion

The Bland-Altman plots show that the October 4, 2019 version of the HeartBeat algorithm, used in conjunction with a Contec CMS50EW pulse oximeter, MediBeat app, and a Samsung Galaxy XCover 4 smartphone, was not sufficiently accurate to meet ESH-IP2 standards for measuring the blood pressure of adults in the general population; therefore, as per the protocol, it is not recommended for personal or clinical use in this configuration.

## Appendix

Multimedia Appendix 1 Information given during recruitment.

## Abbreviations

ESH-IP2: European Society of Hypertension International Protocol revision 2010
LOA: limits of agreement
PPG: photoplethysmogram
SpO_2_: peripheral capillary oxygen saturation
