# Supplementary material for: Web-Based Smartphone Algorithm for Calculating Blood Pressure From Photoplethysmography Remotely in a General Adult Population: Validation Study
Source: J Med Internet Res. 2021 Apr 23;23(4):e19187. doi: 10.2196/19187 (PMC8105754; doi:10.2196/19187)
Supplement: Multimedia Appendix 1 [file jmir_v23i4e19187_app1.pdf]

# Appendix 1
# Information given during recruitment

## Email recruitment script

**E-mail Subject line:** SE Research – Looking for volunteers for a research study on measuring blood pressure

We would like to invite you to participate in a research study that will investigate a new blood pressure measurement that uses a mobile application developed by HeartBeat Technologies called MediBeat, compared to using the usual method using a blood pressure cuff and stethoscope which is not ideal for self-monitoring or virtual (remote) care.

Your participation would involve having your blood pressure measured by the two methods during one study visit. The new method requires you to wear a finger oximeter device that is approved for use in Canada to measure a finger pulse. The oximeter device will interact wirelessly with the MediBeat mobile application to calculate a blood pressure reading.

Your participation in the study is completely voluntary. You can decide to leave the study at any time by informing one of the clinicians on-site. Your decision to participate, or not, will have no effect on your employment with SE Health.

There are no known or anticipated risks associated with participation in this study.

Attached you will find a copy of a letter of information about the study that gives you full details.

If you are interested in becoming a study participant or have any questions, please contact a member of the SE Health project team, Brianna Croft, at BriannaCroft@sehc.com.

Thank you in advance for your time and consideration.

**Communications SEHC**
E: CommunicationsSEHC@sehc.com
sehc.com | elizz.com
Health

# LOOKING FOR VOLUNTEERS
# FOR A RESEARCH STUDY ON
# MEASURING BLOOD PRESSURE

This study will compare the accuracy of using new technology compared to the traditional blood pressure cuff method.

A total of 45-60 minutes of your time is required.
Your blood pressure will be taken 7-9 times.

Whether you volunteer or not has NO EFFECT on your employment at SE Health. Participants will receive a $50 gift card as compensation for their time.

Interested? Please email Brianna Croft at BriannaCroft@sehc.com, who will give you a call to tell you more, answer your questions, and arrange the time to participate

Research
Centre

# LOOKING FOR VOLUNTEERS FOR A RESEARCH STUDY ON MEASURING BLOOD PRESSURE

This study will compare the accuracy of using new technology compared to the traditional blood pressure cuff method.

**A total of 45-60 minutes of your time is required.**
**Your blood pressure will be taken 7-9 times.**

Whether you volunteer or not has NO EFFECT on your care at this clinic or from any health care provider. Participants will receive a $50 gift card as compensation for their time.

Interested? Please take one of the attached forms, sign it, and hand it to a nurse here in the clinic. One of the study team members from SE Health will give you a call to tell you more and answer your questions.

Research Centre

# PARTICIPANT INFORMATION AND CONSENT FORM FOR RESEARCH

**Observational trial: Determining the accuracy of the HeartBeat Algorithm for calculating blood pressure**

You are being invited to participate in a research study that will investigate a new blood pressure measurement that uses a mobile application developed by HeartBeat Technologies called MediBeat, compared to using the usual method using a blood pressure cuff and stethoscope which is not ideal for self-monitoring or virtual (remote) care.

This study is being conducted by Drs. Paul Holyoke and Margaret Saari from the Saint Elizabeth (SE) Research Centre and is funded by the Centre for Brain Health and Aging Innovation.

<u>What we are inviting you to do</u>
Your participation would involve having your blood pressure measured by the two methods during one study visit. The new method requires you to wear a finger oximeter device that is approved for use in Canada to measure a finger pulse. The oximeter device will interact wirelessly with the MediBeat mobile application to calculate a blood pressure reading.

During the study, your nurse will collect some basic information about you such as your age, and any current medications. This information will be used to help validate the new blood pressure measurement method.

Your blood pressure will be measured 4 times manually with a blood pressure cuff and 3 times with the wearable finger pulse oximeter at your study visit. The study visit will take approximately 45 minutes of your time. You will receive a copy of your current blood pressure results from the manual blood pressure measurement for your own information.

<u>Is participation in the study voluntary?</u>
Your participation in the study is completely voluntary. You can decide to leave the study at any time by informing one of the clinicians on-site. Your decision to participate, or not, will have no effect on your relationship with SE Health (including your employment if applicable) or your quality of care.

<u>Risks and benefits</u>
There are no known or anticipated risks associated with participation in this study. Please see the section about voluntary participation for more details.

<u>Are study participants to be paid in this study?</u>
To thank you for giving up your valuable time to help out on this study, you will receive a $50 pre-paid VISA gift card.

<u>Will my identity be known to others?</u>
If you agree to participate in the study, any documents with your name will only be seen by a member of the research team. Any study results which are reported will not identify you by name or contain any identifiable information.

Will the information I provide to the researchers be kept confidential?
If you decide to participate in this study, the research team will only collect the information they need for this study. Records identifying you will be kept confidential and, to the extent permitted by the applicable laws, will not be disclosed or made publicly available, except as described in this consent document.

All aspects of the study will be confidential and only the investigators and research staff will have access to information collected. All paper-based information will be securely locked in filing cabinets at the SE Research Centre. Electronic data will be stored on secure servers owned by SE Health Care, which comply with the standards of the Personal Health Information Protection Act. All records (paper and electronic) will be securely destroyed within 7 years.

Ethics review
This study has been reviewed by the Southlake Regional Health Centre Research Ethics Board (an independent committee that reviewed the ethical aspects of this study to help protect the rights and welfare of study participants). If you have any questions regarding your rights as a study participant or about ethical issues related to this study, please contact the Chair of the Ethics Board, Dr. Pravesh Jugnundan, at 905-895-4521 ext. 6638.

Is there a conflict of interest?
There are no conflicts of interest to declare related to this study.

Who should I contact if I have questions regarding this study?
If you have any questions regarding this study or would like additional information to assist you in reaching a decision about participation, please contact Elizabeth Kalles, Research Assistant from the SE Research Centre team, by phone at 416-459-5095 or by email at ElizabethKalles@sehc.com.

**Consent of Participant**

By signing this consent form, you are not waiving your legal rights or releasing the investigator(s) or involved institution(s) from their legal and professional responsibilities.

______________________________________________________________________________

☐ I understand the information contain in this consent.

☐ I have had the opportunity to ask any questions related to this study, to receive satisfactory answers to my questions, and request any additional details I wanted.

| | |
|---|---|
| ______________________________ | ______________________________ |
| Print Name | Date and Location |
| ______________________________ | ______________________________ |
| Signature of Participant | Witnessed |
